# Worker Preferences for a Mental Health App Within Male-Dominated Industries: Participatory Study

**DOI:** 10.2196/mental.8999

**Published:** 2018-04-25

**Authors:** Dorian Peters, Mark Deady, Nick Glozier, Samuel Harvey, Rafael A Calvo

**Affiliations:** ^1^ Wellbeing Technology Lab Faculty of Engineering and IT University of Sydney Sydney Australia; ^2^ Black Dog Institute Sydney Australia; ^3^ School of Psychiatry University of New South Wales Sydney Australia; ^4^ Brain and Mind Centre University of Sydney Sydney Australia

**Keywords:** mental health, mhealth, mobile apps, workplace, men, participatory design

## Abstract

**Background:**

Men are less likely to seek help for mental health problems, possibly because of stigma imposed by cultural masculine norms. These tendencies may be amplified within male-dominated workplaces such as the emergency services or transport industries. Mobile apps present a promising way to provide access to mental health support. However, little is known about the kinds of mental health technologies men would be willing to engage with, and no app can be effective if the intended users do not engage with it.

**Objective:**

The goal of this participatory user research study was to explore the perceptions, preferences, and ideas of workers in male-dominated workplaces to define requirements for a mental health app that would be engaging and effective at improving psychological well-being.

**Methods:**

Workers from male-dominated workplaces in rural, suburban, and urban locations took part in an exploratory qualitative study involving participatory workshops designed to elicit their perspectives and preferences for mental health support and the design of an app for mental health. Participants generated a number of artifacts (including draft screen designs and promotional material) designed to reify their perceptions, tacit knowledge, and ideas.

**Results:**

A total of 60 workers aged between 26 and 65 years, 92% (55/60) male, from male-dominated workplaces in rural (16/60, 27%), suburban (14/60, 23%), and urban (30/60, 50%) locations participated in one of the 6 workshops, resulting in 49 unique feature ideas and 81 participant-generated artifacts. Thematic analysis resulted in a set of feature, language, and style preferences, as well as characteristics considered important by participants for a mental health app. The term “mental health” was highly stigmatized and disliked by participants. Tools including a mood tracker, self-assessment, and mood-fix tool were highly valued, and app characteristics such as brevity of interactions, minimal on-screen text, and a solutions-oriented approach were considered essential by participants. Some implementation strategies based on these findings are included in the discussion.

**Conclusions:**

Future mental health mobile phone apps targeting workers in male-dominated workplaces need to consider language use and preferred features, as well as balance the preferences of users with the demands of evidence-based intervention. In addition to informing the development of mental health apps for workers in male-dominated industries, these findings may also provide insights for mental health technologies, for men in general, and for others in high-stigma environments.

## Introduction

Men account for 75% of suicides but are less likely to seek help for mental health problems and are more likely to use unhelpful coping strategies [1]. Low rates of help-seeking in men are likely due in part to the stigma imposed by dominant cultural masculine norms that discourage emotional expression and help-seeking as signs of weakness [2-4]. This effect and its impact on mental health are likely to be amplified within hyper-masculinized cultures such as male-dominated workplaces [5]. Furthermore, some male-dominated industries, such as emergency services, mining, and transport, involve additional risks and obstacles to mental health, including isolation (in rural and remote locations) and increased exposure to traumatic incidents [6].

Owing to the prevalence of mobile phone use, mobile phone–based mental health interventions have become an increasingly popular approach to overcoming traditional barriers to access of mental health services [7]. However, an app can only be effective if it is engaged with by the population it is intended for, which means knowledge of user perceptions, values, and preferences with regard to app-based support is critical.

Participatory design, as a methodology for involving users in the design process, provides a set of methods and user-centered orientation for eliciting user perspectives, preferences, and ideas for the codesign of technologies [8]. Participatory design is also increasingly used as a method for the design of health and mental health technologies to empower patients or end users by involving them in development and to ensure tools are more likely to be engaging and effective [9-11]. Furthermore, participatory methods provide a way to create a democratic and destigmatized space in which to discuss mental health issues. Previous qualitative studies have shown that when men are provided with relevant information in a safe space, they can be willing to discuss mental health issues [10].

In this paper, we present results from the user research phase of the development of an app intended to improve the well-being of workers in male-dominated workplaces. A participatory design approach was selected with the intention of gleaning insights that would help guide the design in ways that avoided triggering stigma (ie, via language and style) and that would ensure elements of clear value for users would be built into the app to increase uptake and ongoing engagement.

Our aim was to help address some of the difficulties in engaging men with mental health support by (1) presenting qualitative results of their perceptions, preferences, and ideas related to mental health technologies and (2) providing an example of how a research-based app for mental health might be designed in alignment with these perceptions and preferences. For the latter, we include examples of how conflicts between user requirements and research-based mental health practice might be resolved via interaction design.

## Methods

### Recruitment

Workers were invited to participate in the study via email announcements distributed to employees of 2 male-dominated organizations who were partners on the project: a state fire and rescue service (the seventh largest urban fire service in the world) and a multinational freight transport company based in Australia.

The only eligibility criterion for the study was that participants worked in a male-dominated industry, which was described in the recruitment material as “a largely male industry (eg, transport, fire and rescue, construction).” Before participating in the workshops, participants were provided with an information sheet describing the study and a consent form in alignment with University of New South Wales ethics committee requirements. This research was approved by the Research Ethics Committee at the University of New South Wales (HC16646).

### Participatory Design Workshops

Activity-based workshops (2.5 hours each) were carried out with groups of participants (consisting of a minimum of 5 and a maximum of 17 participants per group). During each workshop, the first author, a user experience specialist, guided participants through a series of activities designed to elicit: (1) their current experience of issues and ideas to do with mental health support in their workplace (a preparatory exercise), and (2) their ideas and preferences for an app that would support mental health for themselves and other workers in male-dominated industries.

Activities were selected to fulfill a series of user research questions defined by the research team and were drawn from the literature on participatory design [8,12,13]. Activities included individual reflection, collaborative ideation, and paper prototyping. Participants were facilitated in generating ideas for desirable functionalities and characteristics and were provided with templates and materials with which to draft screen designs and promotional materials for the app, which elicited preferred use of language and imagery (please see Multimedia Appendix 1 for a detailed outline of the workshop process). Data collected included participant-generated artifacts from each of the activities (ie, filled-in advertisement templates and screen designs), collections of feature ideas on sticky notes, field notes, and audio recordings of each workshop.

**Figure 1:**
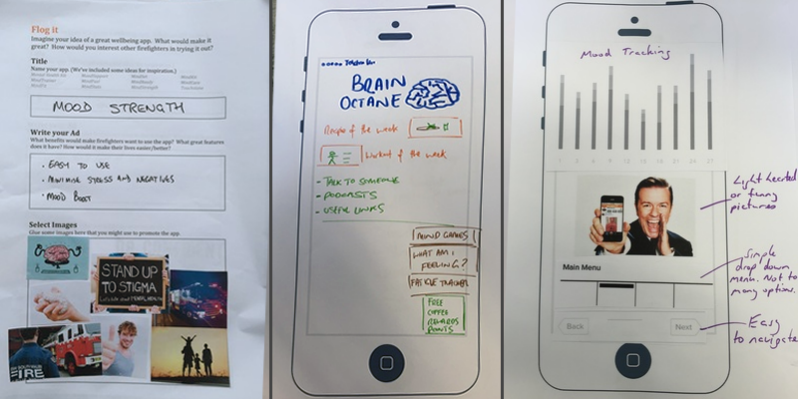
Examples of artifacts generated by participants during workshops (an advertisement template on the left side and 2 screen designs from participants at different workshops on the right side).

In addition to the workshops, a group of 106 workers (different to participants of the workshops) completed a questionnaire intended to collect quantitative information on mobile phone and technology use (results reported in the study by Deady et al [14]). Although this survey focused on separate data, it does provide corroborating evidence for 2 findings emerging from the workshops described herein. First, the survey found that respondents “preferred terms such as ‘well-being’ and ‘mental fitness’ for referring to mental health” (made clear by participants in our workshops). Second, behavioral therapeutic techniques were regarded most favorably (which is also reflected in the feature ideas generated by our workshop participants).

### Data Analysis

Using thematic analysis [15] and consistent with methods for the analysis of generative participatory data [6], the text, imagery from workshop artifacts, as well as verbal transcripts of audio recordings (transcribed using NVivo, a qualitative data analysis software developed by QSR International) were analyzed and categorized into themes. User research was conducted toward a specific outcome (the development of an app), and therefore, analysis was guided by the research questions in support of that outcome. Therefore, of the issues discussed by the participants, analysis focused on those categories of data that would inform app development, namely, themes associated with app features, app characteristics, motivations for engagement, and attitudes relating to language use and visual style that would impact acceptability of the app. Categories were created as feature types, eg, a category “notifications and reminders” included “prompts to retake self-assessment” and “alerts to fresh content in the app.” Initial coding attached labels to text segments which appeared to indicate important material in relation to the research questions. Analysis then progressed iteratively to develop a set of themes that identified sets of features, characteristics, language, and style elements. The text and imagery from workshop artifacts were also incorporated into this thematic analysis (the latter based on the verbal descriptions given the images by participants).

Figure 1 shows examples of the types of artifacts generated by participants during workshops.

## Results

### Participatory Design Workshops: Demographics

A total of 60 workers (mean age 47 years; range 26-65; 55/60 or 92% male) participated in one of the 6 workshops conducted between February and April 2016. Of these, 55 worked for an Australian state fire and rescue service, based at an urban (30/60, 50%), suburban (14/60, 23%), or rural (16/60, 27%) location, whereas 5 worked at a suburban freight transport company. Moreover, 58 (97%) participants reported using a mobile phone.

### Key Features Requested by Participants

Qualitative analysis of 81 participant-generated artifacts (54 screen designs, 27 advertisement templates) together with sets of feature ideas from ideation yielded a set of user-preferred features, functionalities, and characteristics for a mental health app for workers in male-dominated industries. A total of 51 unique feature ideas were generated through these processes (see Multimedia Appendix 2). Among these, 7 emerged independently and consistently in all workshops:

- Mood tracking
- Mood-fix (for quick fixes of negative mental states)
- Easy access to urgent help
- Links to mental health support organizations (external and employer-provided)
- Guidance on how to deal with specific situations that occur on the job
- Stories of lived experience from respected members of the community
- Notifications and reminders to use the app

Seven more features emerged independently in at least 4 workshops.

- A self-assessment tool
- Solutions or strategies based on the self-assessment
- Something to show your progress
- Guidance on how to have mental health discussions (with friends/family)
- Brain games or puzzles
- A dashboard (showing progress and stats)
- Notifications alerting to fresh content

Below, we elaborate on 6 of these in greater detail including the mood tracking tool, mood-fix tool, guidance on mental health discussions, stories of lived experience (the 4 among the top 7 that warrant further explanation), and self-assessment (because it was a feature important to both participants and researchers).

#### Mood Tracking

**Figure 2:**
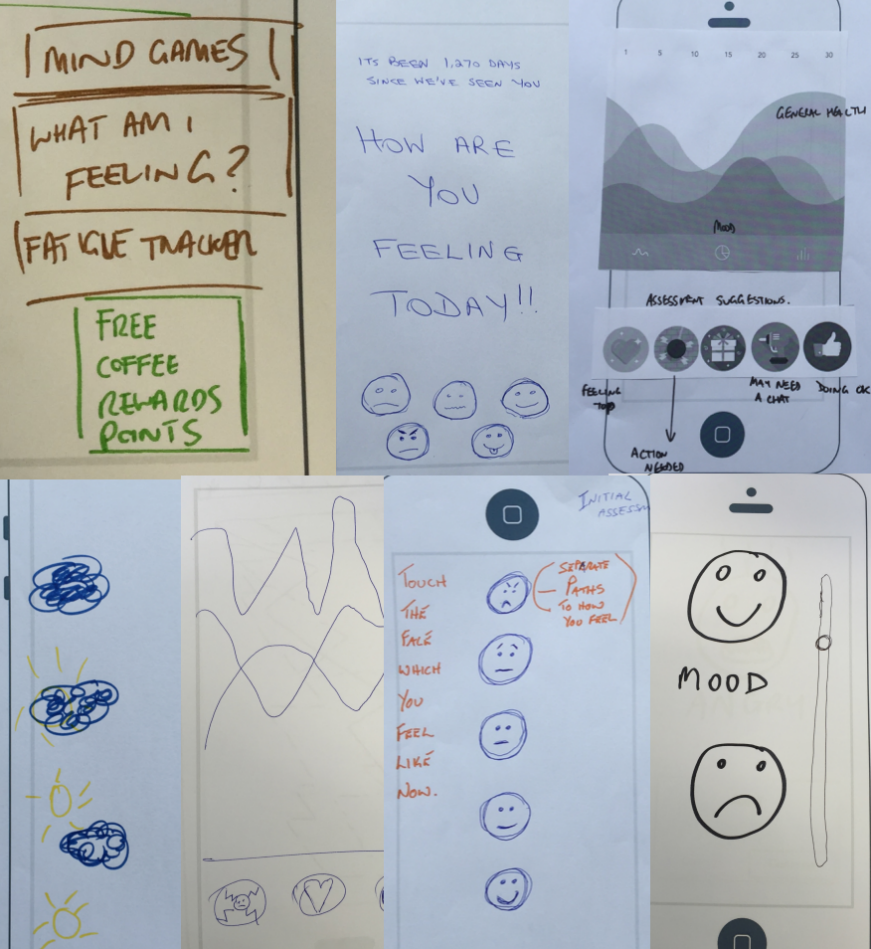
Seven different instances of a mood tracking feature generated independently by participants from different workshops.

The idea of mood tracking emerged in all the 6 workshops. Figure 2 shows some of the various ways in which mood tracking was envisioned by participants as part of workshop activities.

Specific ideas about design implementations of the mood tracker varied widely, with some participants reporting they would only use a very simple tracker (eg, a set of smiley faces), whereas others suggesting the app should provide the ability to label one’s emotions in detail to improve self-awareness. Some requested the ability to record reasons for current moods, whereas others wanted to link moods to activities such as sleep and exercise. There were also varied perspectives on whether mood logging should occur once a day, more often, or at random times. The following quotations demonstrate some of the ways participants expressed a desire for mood tracking:

So that people can have visual feedback at the end of every week, month, cause most people won’t track back, they’ll just go, “I feel like shit,” but they won’t necessarily track back and go “this is why I feel like shit” …When you get visual things you might go “Oh, well actually, maybe I’ve had a lot of stuff going on--maybe it’s not that weird that I’m not feeling great now.”

How you’re feeling, what your sleep’s like and you can track that.

I wouldn’t do it once a day, I’d probably only do it if I felt shit.

I’m having a great day ’cause this happened or, I’m having a shit day ’cause this happened.

By identifying what emotion they feel, because most people aren’t actually sure what emotion they're feeling. Like “Oh, am I disappointed or am I sad?” The process of having to identify, that's actually what I feel—because a lot of people are not happy or sad or angry. That’s the three, there's nothing else.

#### Mood-Fix Tool

Participants highly valued the notion of a tool that could be used for instant relief in moments of distress, and they described this in various ways including a “mood boost,” “stress button” help to “reset your mind,” or a tool to “change focus”:

Mood boost…trying to get yourself back into a happy place.

Just buttons you can press on. Stress relieving games, stress relieving ideas, ideas to try and change your train of thought.

#### Self-Assessment Tool

There was strong interest among participants in having a way to judge one’s own state of mental health, with some participants highlighting the difficulty some people have in realizing they need help:

Hard hitting questions to identify whether your mind is where you think it is.

Quick stress check that identifies you may require help.

Over here is a bit of a resilience status, so maybe you could gauge…how resilient you are to mental health issues.

For the assessment bit it’s “how are you feeling? Are you feeling happy, sad, fatigued, mood swings?” You can self-assess and see how you’re going and that can allow you to progress to the next stage in sorting things out.

Sometimes, you don’t realize you’re in that position until someone drags it out of you, so I don’t know if there’s a way to identify that you’re not yourself.

#### Guidance on Having Mental Health Discussions

Participants expressed a desire for help in determining how they could discuss mental health with others and how they might help someone they suspected of struggling with a mental health issue. Specifically, they requested tips on ways to talk to family about mental health and on how to help a friend:

What if, as part of the app, you had coaching techniques on how to guide conversations, how to bring things up very casually—it could be for your own benefit, like if I need to talk about this, how do I raise that with my crew? how do I raise it with my family? But also if you can see in one of your workmates, or one your family members, someone is struggling, how do I raise that with them and put them in a position where they feel comfortable talking to me about it or where they feel open and trusting of me to express how they’re feeling? That would be really useful.

#### Tackling Stigma Through Personal Stories

Participants were openly aware of the problem of stigma within their population and proposed various possible solutions in relation to technology design. First, they reported that selection of terminology would be very important as some language carries a high stigma burden (as described further below). Second, participants across workshops independently recommended the use of personal stories of lived experience (preferably in the form of videos) as an effective method of reducing stigma, in particular, if the personal stories are told by respected members of the public or celebrities:

Find cool people that have mental health issues.

Sometimes if someone’s gone through something and they’re willing to admit to it and go on to show how it affected them and how they got through it, that’s a good way ’cause you can relate to that.

#### Language and Style Preferences

Many of the preferences expressed by participants are related to language, style, or technical characteristics (ie,“easy-to-use,” “highly visual,” and “accessible off-line”) rather than features or functionalities. These characteristics are included below:

I like graphic sort-of-things. On our Intranet we have two screens: one is all the details, the second screen is a graphic representation of what the watch desk is and I go there all the time--it’s a lot easier for me to go to that and click on what I need.

I find it frustrating reading too much information—long-winded, too wordy, what are you trying to say? Push the button, tell me! I don’t really feel like reading. I’m not feeling well, do you really want me to work at it, as well?

There was apparent consensus among participants that the term “mental health” equated to serious illness, being “crazy,” and was highly stigmatized and something many would not want to be associated with. In contrast, the terms “wellbeing,” “resilience,” and “mental fitness” were perceived as more positive and therefore preferable:

I think the more serious you make the issue, the more stigma there is attached to it. Like if you break down and you can have guys talking about it on a colloquial level, then more people are going to be likely to engage with it. If you make it out to be a very like “oh, he has mental health problems, that’s a big deal” people are going to step away from it in a big way.

We just like the word MindFit. People seem to be using fitness apps, and it’s got the same word… It’s got a positive connotation—nothing negative about it.

Mental health goes hand in hand with physical fitness to a certain point of view ’cause while exercise is probably one of the best things you can do if you’re struggling with mental health… but if you get some sort of link, like an offshoot of the fitness thing…So, you go, “I’m gonna do my 4K run in 11 mins and then going around the track and getting better” but it’s also linked to your mental health app.

In conversation, participants themselves opted for these terms to describe mental health issues.

Negative terms used by participants were as follows:

- Stress or feeling stressed
- Struggling
- Not yourself
- Feeling shithouse, having a shit day
- Depressed
- Feeling a bit “How ya’ going”
- Having negative thoughts and behaviors

Positive terms used by participants were as follows:

- Change your mindset
- Relax and release
- Just get it all out
- Mood boost

### Motivators for Mental Health Self-Care

The 3 most salient motivators for either help-seeking or proactively maintaining mental health were concern for family, preparedness or prevention (preventing problems before they occur and being prepared for psychologically challenging situations), and social influence.

In terms of the family as motivator, there was a widely expressed concern to do with one’s own stress and mental health having a negative impact on the family and a desire not to take work stress home but to be one’s best self for one’s family:

Trying to get yourself back in a happy place, especially if you have a young family, as we said before, any negative thoughts are going to impact negatively on the family as well.

With regard to preparedness, many participants (particularly those who worked in first responder settings) expressed a strong interest in proactively strengthening resilience as a component of overall health to prevent mental health problems and to be prepared for psychologically challenging situations:

[The app’s] not for somebody who's in the process of severe mental problems. This is more along the lines of getting it before it happens.

More resilience coaching and training before you have issues… to prepare you for what you’re doing.

What can we do to build resilience?...Like how do we get in before a major issue?

Finally, participants also reported being influenced by the attitudes and behaviors of friends and coworkers in relation to technology use:

Most of the apps I use have been recommended by one of our mates…

If your mate is a bit overweight and they started using Strava, and they got addicted to it, and you say “he lost 10 kilos and is riding 100 K's every morning; gee he’s doing alright, maybe that’s helped him do that.” So probably results from something is going to make you share it.

More broadly, participant data revealed a preference for a practical and active solutions-based approach. For example, participants commonly requested “tips,” “strategies,” “tools,” “exercises,” “direct links to solutions,” and suggested actions that can be taken:

The guide might say “you’re a low risk, do this” or “you’re a high risk, do something else.”

## Discussion

### Principal Findings

This participatory user research study aimed to explore the preferences of those in male-dominated industries with regard to mental health mobile phone apps. Given the well-documented difficulties engaging workers from these types of industries in mental health promotion, such insights are likely to be crucial to the successful development of new e-mental health interventions. Our results highlight that this group of workers does have a set of features, language, and style preferences that they feel are essential in any future mental health app. The term “mental health” was highly stigmatized, with alternative positive expressions for mental health, like “mental fitness,” being greatly preferred. A range of specific features including mood tracking, self-assessment, and “mood boost” tools were highly valued in addition to characteristics such as brevity, minimal on-screen text, and a solutions-oriented approach.

Men have traditionally poor rates of mental health help-seeking among face-to-face services and poor engagement with health promotion activities more generally, although relatively little work has focused on the specific preferences of this group regarding eHealth technologies for mental health. Recent studies reporting on young men’s attitudes and behaviors in relation to mental health and technology allow for comparison with the perspectives and preferences identified among our participants [16,17]. Ellis et al [16] found that young men shared a preference for action-oriented strategies (rather than talk-based strategies) for self-help. In addition, the adolescent and young male participants expressed interest in content featuring celebrities or role models, just as our cohort endorsed the use of respected community members as a strategy to battle stigma. Finally, Ellis et al report a broad interest among young men in tools to help “identify, discuss and manage mental health issues” also shared by the participants in our study. In spite of these similarities, our cohort of working aged men (compared with Ellis’ young study population) expressed a number of additional concerns. For example, the adults in our population expressed motivational drivers and concerns stemming from being the head of a family or in a “caretaker” role. The adults in our study also demonstrated a greater diversity with regard to technology use owing both to some older participants having less experience and less free time to engage with mobile technology. Finally, although Ellis et al report that many young men “tended to believe they would never be personally affected by a mental health difficulty,” this was not prevalent among our adult male cohort where participants demonstrated significant lived experience of mental health issues, either personally (sometimes due to work-related incidents) or secondhand via experiences of friends’ or family members’ struggles.

### Translation of Findings Into Design of a Mental Health App

We report on some of the ways we translated the insights from user research into app design, including how we resolved issues of misalignment between user and researcher requirements. Results from user research highlighted the importance of including features of clear and practical value to the users themselves. Interestingly, these features only minimally overlapped with those deemed important by many mental health researchers, and our approach to reconciling the 2 is discussed below.

Evidence-based programs for mental health support generally require lengthy and reflective sessions of interaction with a therapist or with therapeutic content generally consisting largely of written text. In contrast, our participants reported that they would not want to spend more than a few minutes at a time with a mental health app, and that an appealing app should require as little text reading as possible. Furthermore, rather than programs of self-improvement, they were largely interested in self-tracking, self-assessment, as well as strategies and tools for problem solving in the moment of need (a solutions-based task-oriented approach).

The research team took 2 key approaches to resolving misalignment between user preference and clinical need. First, we incorporated 3 of the top user-preferred features into the app (mood tracking, mood fixing, and self-assessment). One of these (self-assessment) aligned with clinical requirements for the app, and the other 2, although not specified as clinically necessary for the app, are components commonly used in evidence-based interventions, such as cognitive behavioral and behavioral activation therapy [18]. Second, we redesigned the evidenced-based therapeutic content to allow it to meet user requirements for brevity. We describe these approaches in detail below and present them as examples of how a mobile phone app for mental health might be designed to meet user needs. The app has been made available and can be downloaded from both the Apple and Google Play app stores [19].

**Figure 3:**
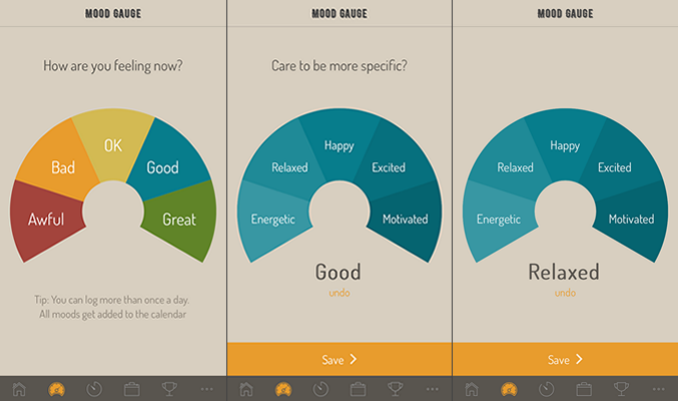
The mood tracker allows users to log a mood based on a very simple 5-item scale and also provides the opportunity for those who are interested in being more specific to reflect on a more specific emotion label. Both use cases emerged from varying participant perspectives at workshops.

With regard to a mood-tracking feature, our results indicated that some users wanted to be able to enter specific emotion labels, whereas others insisted they would only use a mood tracker if it were very basic and easy to use. Our proposed design solution, therefore, includes both options in a 2-step process. On the first screen, the user can select from 5 simple mood labels (awful, bad, ok, good, and great). On the next screen, the user can choose to “be more specific” by selecting from 5 additional specific emotion labels that elaborate on the mood already selected or they can ignore this option and just click “save” (see Figure 3).

**Figure 4:**
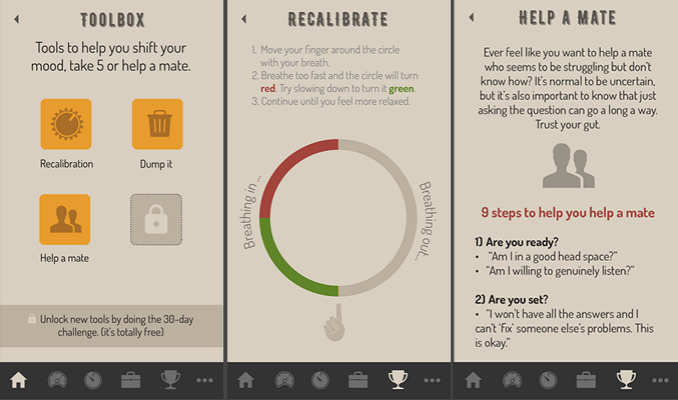
An example of how learned prevention skills and techniques could be presented to users within a mental health mobile phone app.

The desire for a “Mood fix” feature was translated into a “Toolbox” that could act as a container for learned prevention skills and techniques an individual acquires. For our implementation, the “Toolbox” could hold a number of different approaches to managing negative moods as well as tools for other topics mentioned in workshops such as guidance on how to help a friend who is struggling. Finally, the toolbox allowed a series of new tools to be earned as rewards for moving through the therapeutic core of the app, described below. An example of how a toolbox may look is provided in Figure 4.

**Figure 5:**
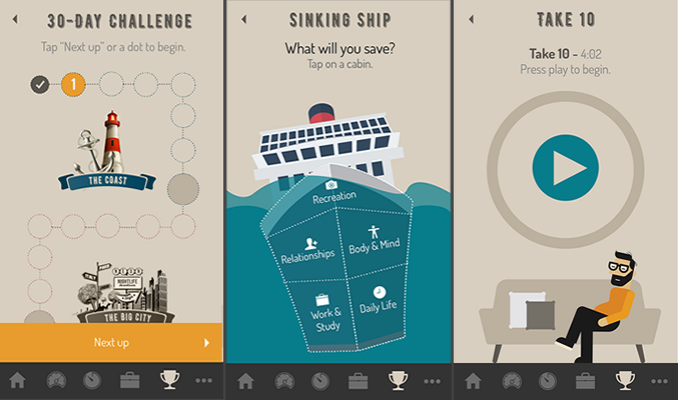
An example of how traditional interventions might be broken into 2- to 5-min sessions delivered over a longer period.

A key misalignment between the findings of our study and the need for more detailed, lengthy therapeutic interventions is users’ desire for brief interactions. The solution we propose for this is to restructure established interventions into small chunks of highly visual interactive content and single exercises that could be undertaken by users once a day (in 2- to 5-min sessions) over a longer period. In this way, longer evidence-based interventions can be translated into “Daily challenges.” Figure 5 shows an example of our approach: a 30-day program of evidence-based interventions or “Daily challenges,” which included exercises based on behavioral activation theory, mindfulness, and psycho-education.

### Limitations

Consistent with Brownhill [20], we found that when given a safe space in which to discuss mental health issues, participants were willing to discuss these issues openly and were highly generative of ideas. However, it should be noted that this is also likely to have been influenced by a self-selection bias in that those who volunteered for the study are likely to represent those more willing to discuss mental health issues. We did not collect information on each participant’s previous experience with mental health issues (eg, by proxy, lived experience) or to what extent each participant identified with a strong concept of masculinity. We also acknowledge that there will be limitations related to our particular applied focus to the data analysis. Data were categorized specifically to inform the development of an app, and other approaches to analysis would likely shed light on other aspects of the data. The participant cohort was also limited with respect to geography and the variety of industries represented. The vast majority of participants were drawn from the emergency services (specifically fire and rescue), and all participants were from Australia, meaning their responses may not be generalizable to other populations. For example, other aspects of emergency service work, apart from being male-dominated, may have influenced the results obtained (eg, that the work is nonsedentary, high-risk, and involves exposure to traumatic incidents).

### Conclusions

In this study, we attempted to address the gap in understanding of male preferences for mental health apps, in light of the high stigma and lower help-seeking behavior associated with men. Results from our user research highlighted the importance of using nonstigmatized language and including features of clear and practical value to the users themselves. Most saliently, the term “mental health” was considered highly stigmatized and should be avoided, especially when the focus is preventative or promotional rather than for treatment. In addition, assessment and ongoing tracking of mood and mental state were highly valued as were highly visual and solution-oriented strategies and tools for fixing problems. Further research involving men from other countries and within other industries would contribute to generalization of these results.

## Appendix

Multimedia Appendix 1 Design outline for participatory workshops.

Multimedia Appendix 2 Feature ideas generated via participatory design activities.
