# Supplementary material for: Worker Preferences for a Mental Health App Within Male-Dominated Industries: Participatory Study
Source: JMIR Ment Health. 2018 Apr 25;5(2):e30. doi: 10.2196/mental.8999 (PMC5943624; doi:10.2196/mental.8999)
Supplement: Multimedia Appendix 1 [file mental_v5i2e30_app1.pdf]

Design Outline for Participatory Workshops

Aim of project: To develop a web-based support tool that will help workers in male-dominated workplaces to: 1) better understand their mental health risk, 2) better manage and respond to mental health issues.

Activity/Method	Min	Description	Materials	Artefacts	Outcomes
**Opener**	15	1. Ask everyone to say their name and mention an app they like or use frequently.<br>2. Summary of why we're here, their expertise, and what they'll be co-designing/contributing to.	Flipchart, markers, stickies	List of favourite apps	
**Mental health and firefighters** (customisation of the 4C's method)	30	Participants are asked to add ideas using sticky notes to each of four quadrants on a chart: 1. Challenges, 2. Supports/Strengths, 3. Ideal/Objective, 4. Who's involved/effected).<br><br>In the last 15 minutes, results are clustered and discussed in the larger group.	Flipchart, markers, stickies.	Chart of perspectives on different angles of the problem of mental health demonstrating various aspects of the issue & language use.	• Personal identity as a firefighter<br>• How people think about risks and risks to their health,<br>• Attitudes towards stress,<br>• What do they take into account with their health<br>• What things do they consider changing?<br>• Language they use around stress/mental health;<br>• How to communicate risk factors in a language that they will appreciate.

**Heart Hand Mind**	30	Ask them to envision an app that they would actually be interested in using to get support for stress/mental health issues or goals<br>**Mind**: What would make it effective and useful;<br>**Hand**: What would make it practical and convenient;<br>**Heart**: what would make it emotionally engaging.<br>They write their ideas on stickies and add them to the board. Then ideas are discussed.	Pre-printed Poster with spaces for heart/hand/mind for the posting of ideas; sticky notes	Poster with a list of clustered ideas generated by participants	• What is a meaningful way to feed back information to them about risk (percentage? visual?<br>• Preferences for conceptualisation of a mental health/mental wealth/wellbeing app<br>• Preferred features, (eg. team competitions)
**Flog it**	30	In small groups, each group creates a draft advertisement for the app, revealing values, attractions and perceived benefits. (20 min)<br>Each group presents their ad. (10min)	3 packs of: Crafting materials such as magazines, images, glue and markers. Include various style graphics from apps, video games and websites. Also include variations on terms on cards. =	1 co-created ad collage for each group showing perceived benefits, features and effective communication approaches to audience.	• Preferred features,<br>• Preferences for App communication<br>• Language they use around stress/mental health;<br>• Preferences for the look, feel, and attitude of a wellbeing app
**"Design the app" (wireframing)**	30	In small groups, participants draw (or paste with provided screen objects) a number of screens of the app to show	Prototyping cut-outs (buttons, frames, sliders, etc.), mobile	A series of user-created screen wireframes	• Preferred features<br>• Preferences for App communication<br>• Language they use around

		how it would work and what features it might have. (20min) Groups then describe their screens (10 min)	screen templates, pens and markers.		stress/mental health;
**Tips for the designers – closing**	10	Ask participants what advice they would give to the designers and developers of this app. Collate onto board.			
**Total Duration (hours)**	2.5 hrs				

Space Requirements

Location options

- Go to them, eg. station - more convenient for them but may bias response by associating it with the organization)
- A union venue (may help reinforce intentions to support and include them)
- Community venue near a station
- University (potentially free but may be less convenient for them, and not on their turf)

Room requirements

- **Campfire**: Area where a circle of seats can be made for whole group discussion and sharing.
- **Breakout tables** - where small groups can collaboratively write and work.
- **Walls for hanging** – Easily accessed walls on which flipcharts and posters can be posted with blue-tac. Enough space in front of the walls for participants to huddle around.
- **Fuel station -** Tables for food/drinks

Materials (6-9 participants)

- Flipcharts with stand.
- Thick Markers – a box for each small group + 1 for facilitators (4)
- Pens (9)
- Sticky notes - 1 block per participant (9)
- A variety of magazines/Images (eg. clipart) for "Flog it"
- Feature & Term Cards (for "Flog It" to test terms and features we're considering)
- Scissors (3)
- Glue (3)
- Pre-printed Poster with spaces for heart/hand/mind for the posting of ideas
- Magnetic Prototyping kit
- Fuel station: Candy, mints, water, juice, fruit, crackers, trail mix (beer?).
