# Supplementary material for: Worker Preferences for a Mental Health App Within Male-Dominated Industries: Participatory Study
Source: JMIR Ment Health. 2018 Apr 25;5(2):e30. doi: 10.2196/mental.8999 (PMC5943624; doi:10.2196/mental.8999)
Supplement: Multimedia Appendix 2 [file mental_v5i2e30_app2.pdf]

Appendix 1: Feature ideas generated via participatory design activities

**Feature theme**	**Feature**
**Tools for relieving stress in the moment**	
	Mood-fix tool
	Guided meditation/breathing exercises
	Games/puzzles
	Ability to upload photos to cheer you up
	Techniques to modify negative thoughts/behaviour
	A way to vent
**Health and Fitness**	
	Recipes for mood “Food for thought”
	Info on the effects of food on mental wellbeing
	Workouts / exercises (on video)
	Stats on your fitness
	Sleep tracking
	Information on Work, Health, and Safety
**Mood Tracking**	
	Mood tracker (ability to track moods over time)
	Ability to record the causes of or activities triggering moods
	Graphs of Mood over time to show trends
**Assessment and diagnostic tools**	
	Self-assessment quiz with feedback (to indicate current state of wellbeing/mental health)
	Suggestions for what you can do based on self-assessments
	List of signs and symptoms to look out for (for different mental health problems)
	Help with assessing someone you’re concerned about
	Tracking of multiple of assessments to progress how you’re doing over time.
**External links**	
	Links to mental health organisations
	Links to employer-provided resources (eg, Chaplain, Employee Assistance Program)
**Social features**	
	Chat with a professional
	Connect to other users within the app (eg, dedicated forum)
	Way for family to check in on you throughout the day
	Connection to people who have opted to be a resource for others (experienced peers).
	Ability to like/share a section you find useful

**Testimonials & endorsements**	
	Facility for people to provide and view testimonials of the app
	Endorsement of app by respected individuals.
**Psycho-education and coping strategies**	
	Guidance on how to deal with specific situations that occur on the job (traumatic work incidents, difficult members of the public, death, injury)
	Guidance on how to have mental health discussions (with friends/family)
**Personalisation and progress**	
	Personal information that allows app to be customised.
	A dashboard that shows progress (eg, quiz stats, exercises completed)
	Challenges and goals
	Ability for the app to know when you're at work/on shift.
**Physiological sensors**	
	Heart rate monitor
	Voice recognition of stress
	Facial recognition of stress
**Notifications and reminders**	
	Prompts to retake self-assessment regularly (eg, "it's been x days since your last check-in.")
	Alerts to fresh content in the app (egg, new exercises, games, videos)
**Ease-of-use features**	
	First time user guidance
	Help on how to use the app
	Voice control (for use while driving)
	Adjusts for orientation of phone)
**Other**	
	Brain training
	Easy access to emergency help.
	Stories of lived experience (preferably video)
	Evidence that you're no alone (eg, 1 in 5 are feeling this way/have depression")
	Inspirational quote or thought of the day
	Facts and trivia
	List of ideas for what to do on your work breaks for rest
